# Association of Medicare-Medicaid Dual Eligibility and Race and Ethnicity With Ischemic Stroke Severity

**DOI:** 10.1001/jamanetworkopen.2022.4596

**Published:** 2022-03-31

**Authors:** Pamela R. Bosch, Amol M. Karmarkar, Indrakshi Roy, Corey R. Fehnel, Robert E. Burke, Amit Kumar

**Affiliations:** 1 College of Health and Human Services, Northern Arizona University, Phoenix Biomedical Campus, Phoenix; 2 Department of Physical Medicine and Rehabilitation, School of Medicine, Virginia Commonwealth University, Richmond; 3 Sheltering Arms Institute, Richmond, Virginia; 4 Center for Health Equity Research, Northern Arizona University, Flagstaff; 5 Department of Neurology, Beth Israel Deaconess Medical Center, Harvard Medical School, Marcus Institute for Aging Research, Boston, Massachusetts; 6 Division of General Internal Medicine, University of Pennsylvania Perelman School of Medicine, Philadelphia; 7 Center for Health Equity Research and Promotion, Corporal Crescenz VA Medical Center, Philadelphia, Pennsylvania

## Abstract

**Importance:**

Black and Hispanic US residents are disproportionately affected by stroke incidence, and patients with dual eligibility for Medicare and Medicaid may be predisposed to more severe strokes. Little is known about differences in stroke severity for individuals with dual eligibility, Black individuals, and Hispanic individuals, but understanding hospital admission stroke severity is the first important step for focusing strategies to reduce disparities in stroke care and outcomes.

**Objective:**

To examine whether dual eligibility and race and ethnicity are associated with stroke severity in Medicare beneficiaries admitted to acute hospitals with ischemic stroke.

**Design, Setting, and Participants:**

This retrospective cross-sectional study was conducted using Medicare claims data for patients with ischemic stroke admitted to acute hospitals in the United States from October 1, 2016, to November 30, 2017. Data were analyzed from July 2021 and January 2022.

**Exposures:**

Dual enrollment for Medicare and Medicaid; race and ethnicity categorized as White, Black, Hispanic, and other.

**Main Outcomes and Measures:**

Claim-based National Institutes of Health Stroke Scale (NIHSS) categorized into minor (0-7), moderate (8-13), moderate to severe (14-21), and severe (22-42) stroke.

**Results:**

Our sample included 45 459 Medicare fee-for-service patients aged 66 and older (mean [SD] age, 80.2 [8.4]; 25 303 [55.7%] female; 7738 [17.0%] dual eligible; 4107 [9.0%] Black; 1719 [3.8%] Hispanic; 37 715 [83.0%] White). In the fully adjusted models, compared with White patients, Black patients (odds ratio [OR], 1.21; 95% CI, 1.06-1.39) and Hispanic patients (OR, 1.54; 95% CI, 1.29-1.85) were more likely to have a severe stroke. Using White patients without dual eligibility as a reference group, White patients with dual eligibility were more likely to have a severe stroke (OR, 1.75; 95% CI, 1.56-1.95). Similarly, Black patients with dual eligibility (OR, 2.15; 95% CI, 1.78-2.60) and Hispanic patients with dual eligibility (OR, 2.50; 95% CI, 1.98-3.16) were more likely to have a severe stroke.

**Conclusions and Relevance:**

In this cross-sectional study, Medicare fee-for-service patients with ischemic stroke admitted to acute hospitals who were Black or Hispanic had a higher likelihood of worse stroke severity. Additionally, dual eligibility status had a compounding association with stroke severity regardless of race and ethnicity. An urgent effort is needed to decrease disparities in access to preventive and poststroke care for dual eligible and minority patients.

## Key Points

**Question:**

Are dual eligibility for Medicare and Medicaid, a proxy for low socioeconomic status, and race and ethnicity associated with stroke severity?

**Findings:**

In this cross-sectional study of 45 459 Medicare fee-for-service patients with ischemic stroke, Black, Hispanic, and dually eligible patients, including White patients with dual eligibility, had significantly increased likelihood of having a severe stroke.

**Meaning:**

Based on these findings, it is important that dual-eligible patients, Black patients, and Hispanic patients receive access to timely and high-quality stroke care to improve stroke recovery and long-term outcomes.

## Teaser

This cross-sectional study examines whether Medicare-Medicaid dual eligibility and race and ethnicity are associated with stroke severity in Medicare beneficiaries admitted to acute hospitals with ischemic stroke.

## Introduction

Stroke-related mortality has declined in the past few decades; however there are striking differences in stroke-related mortality and hospital readmission rates among individuals with low socioeconomic status (SES) and among racial and ethnic minority individuals.^1^ Higher percentages of Black and Hispanic patients survive strokes with greater residual disability compared with White patients, resulting in higher disability-adjusted life years.^2^ Black patients receive fewer treatments recommended by the Get With the Guidelines–Stroke in every region of the United States.^3^ Black patients also have lower access to primary and comprehensive stroke centers that deliver intensive acute and postacute rehabilitation services.^4,5^ Prior studies have found differences in access to recommended stroke treatments, leading to racial disparities in stroke outcomes, including lower physical function abilities^6,7^ and higher rates of 30-day readmission.^8^

Disparities in health outcomes also persist among underserved dual-eligible (DE) beneficiaries enrolled in both Medicare and Medicaid.^9,10,11^ DE is recognized as a proxy for low SES, a social determinant of health.^12,13^ Despite patients with DE and Black patients having a higher incidence and prevalence of stroke^14,15^ as well as higher poststroke residual disability^15^ than non-DE Medicare beneficiaries,^11^ there was previously no information about stroke severity at the time of hospital admission in national Medicare data by race, ethnicity, and SES status. Some evidence suggests initial stroke severity is not associated with race and ethnicity,^16^ but to our knowledge, this has not been investigated in a nationally representative population. Understanding population differences in initial stroke severity will help to find potential solutions for addressing disparities.

Stroke severity can have a strong residual confounding association with patient outcomes, such as 30-day readmission and 30-day mortality. Until October 2016, stroke severity was not reported in Medicare data, but it is now included through the National Institutes of Health Stroke Scale (NIHSS), providing the opportunity to explore these associations. Value-based payment models now incentivize hospitals by patient outcomes, and hospitals have begun to account for the proportion of patients with DE in risk adjustments for hospital readmission reduction programs.^17,18^ Such risk adjustment models may help hospitals that care for a (disproportionately) larger proportion of patients with DE and racial and ethnic minority patients avoid financial penalties.^19^ The purpose of this investigation was to examine the association of DE and race and ethnicity with stroke severity in adults with ischemic stroke after controlling for social risk factors in nationally representative Medicare claims data. We hypothesized that DE status and racial and ethnic minority status would each be associated with more severe stroke impairments at the time of hospital admission.

## Methods

### Ethics and Resource Sharing Statement

The study was approved by the Northern Arizona University institutional review board with a waiver of informed consent owing to the use of secondary deidentified data. We had a data use agreement approved by the Centers for Medicare & Medicaid Services. Interested researchers may replicate the study by obtaining the data and supporting information files from CMS. To reproduce, this study requires 100% Medicare inpatient claims data in the Standard Analytical File (SAF) format, along with other supplementary files described in this section. This study followed the Strengthening the Reporting of Observational Studies in Epidemiology (STROBE) reporting guideline.^20^

### Study Design and Data Source

We used patient-level Medicare claims data in the SAF format, which include administrative claims for all short-term (ie, acute care hospital) hospitalizations of Medicare fee-for-service (FFS) beneficiaries from October 1, 2016, to December 31, 2017. Most patients with stroke are admitted to acute care hospitals for a relatively short stay, (mean length of stay, 5-7 days).^5^ To avoid heterogeneity and bias in our sample, we did not include patients staying in long-term care hospitals because these patients require prolonged, complex medical care, and the average stay is more than 25 days.^21^ The hospital claims file was linked with the Master Beneficiary Summary File (MBSF) and the Chronic Condition Warehouse (CCW) data to retrieve patient characteristics. The CCW was used to determine chronic conditions (eg, previous history of stroke, stroke risk factors, and the behavioral factors of alcohol and tobacco use). The MBSF contains information on beneficiaries’ sociodemographic characteristics, residential location, and program eligibility and enrollment information for parts A, B, C, and D, and DE for Medicaid. Based on prior research on the stroke belt,^22,23,24^ access to preventive care, and social risk factors, we included indicators of social determinants of health and availability of primary care practitioners at the county level, which may be associated with the incidence and severity of stroke. For social determinant of health variables, we obtained the poverty rate and the proportion of the adult population with a high school or equivalent degree from the American Community Survey Census data at the county level. This was linked with Medicare data to obtain these estimates at the patients’ county of residence.^25^ In addition, Medicare data were linked with the 2017 Medicare Provider Utilization and Payment Data to obtain information on the concentration of nurse practitioners and physicians in patients’ county of residence.^26^

### Study Population

The study cohort included FFS Medicare beneficiaries aged 66 years and older who were admitted to acute or critical access hospitals between October 1, 2016, and November 30, 2017, with an admitting diagnosis of ischemic stroke. The diagnosis of ischemic stroke was identified using Medicare Severity–Diagnostic Resource Group (codes 061, 062, and 063) or *International Statistical Classification of Diseases, Tenth Revision, Clinical Modification *(*ICD-10-CM*) codes cited in a previous study.^27^ To obtain prior history of hospitalization, the sample was restricted to patients who had 9 months of continuous enrollment in Medicare FFS (part A and B) prior to hospitalization and 1 month of enrollment following discharge in 2016 to 2017. The eFigure in the Supplement outlines the selection process, which yielded the final analytical sample.

### Independent Variables

Our primary independent variables were DE status and race and ethnicity. DE status was obtained from the MBSF, which includes monthly Medicaid eligibility status. In the Medicare data set, DE was defined as eligibility for Medicaid at any point during the year.^18^ In our study, a patient was classified as DE if they were enrolled in both Medicare and Medicaid at the time of index stroke hospitalization. Using the Research Triangle Institute code from the MBSF,^28^ the race and ethnicity variable was categorized as non-Hispanic White, non-Hispanic Black, Hispanic, and other races.^29^ Individuals listed as Alaska Native, American Indian, Asian, Pacific Islander, other, or unknown were grouped under other for this analysis. Additionally, we created 8 mutually exclusive categories: Black non-DE, Black DE, Hispanic non-DE, Hispanic DE, White non-DE, White DE, other non-DE, and other DE.

### Outcome

The study outcome was stroke severity measured by the claim based NIHSS. In October 2016, the CMS implemented reporting of NIHSS-specific *ICD-10-CM* codes.^30,31^ Hospitals now record and report these data for patients with ischemic stroke as part of the administrative claims data, which will be important for use in risk adjustment and standardization methods to control for patient-level case-mix differences when comparing quality measures between hospitals (eg, 30-day hospital readmission). We calculated the admission NIHSS from *ICD-10-CM* codes associated with acute hospitalization. NIHSS includes 15 items to evaluate the effect of acute cerebral infarction on the levels of consciousness, language, motor strength, ataxia, dysarthria, and sensory loss.^32^ The NIHSS is administered and documented within 12 hours of arrival to hospital in patients with ischemic stroke. We classified NIHSS into 4 categories based on score (0-7, minor stroke; 8-13, moderate stroke; 14-21, moderate to severe stroke; and 22-42, severe stroke), which have demonstrated excellent discriminant properties for 30-day mortality risk.^33,34,35^

### Covariates

We included patient-level sociodemographic characteristics and comorbidity index (ie, the Elixhauser comorbidity score). To control for prestroke risk factors and case-mix differences, we included stroke risk factors (hypertension, hyperlipidemia, diabetes, and obesity) and behavioral risk factors for stroke (tobacco and alcohol use) from the CCW file.^36^ Elixhauser comorbidity indexes include medical conditions based on *ICD-10-CM* diagnostic codes listed in the SAF for the specified acute hospitalization. Since neighborhood SES was recently found to be associated with 90-day poststroke outcomes,^37^ we included poverty concentration rate, proportion of the population with a high school or equivalent degree (low, ≤25%; moderate, 25.1%-30.8%; and high, ≥30.9%), and concentration of nurse practitioners and physicians (low, ≤0.00019 per population aged 65 years and older; moderate, 0.00021-0.00030 per population aged 65 years and older; and high, >0.00030 per population aged 65 years and older) in the county of patient residence to address social risk factors. We used the “Percent of population below poverty” variable at the county-level from the American Community Survey and divided the distribution into tertiles to define low (≤12%), moderate (12.1%-16.4%), and high (≥16.5%) concentration of poverty in a county. Past research has shown a higher stroke mortality rate in the southeastern region of the United States than other regions, called the stroke belt, which could contribute to the disparity in stroke severity.^22^ Using patient residence information from the MBSF, we created an indicator of whether patients are located in counties in the 11 states in the stroke belt: Alabama, Arkansas, Georgia, Indiana, Kentucky, Louisiana, Mississippi, North Carolina, South Carolina, Tennessee, and Virginia. To account for clustering of data by county that might lead to unbalanced sample sizes within clusters, the county was added as a random effect in the model.

### Statistical Analysis

Descriptive statistics were used to characterize the patient attributes, including age, sex, race and ethnicity, DE status, comorbid conditions, stroke risk factors, behavioral risk factors for stroke, and social determinants of health, stratifying by the 4 validated categories of stroke severity (minor, moderate, moderate to severe, and severe). We used χ^2^ tests for categorical variables and *t* tests for continuous variables to test the association of covariates by stroke severity. We reported continuous variables with means and SDs and categorical variables with frequencies and percentages. We estimated multilevel multinomial logistic regression models to analyze the association of DE status and race and ethnicity with the 4 stroke severity categories, using minor stroke as a reference group. In this model, counties were used as a random effect to account for the nested structure of the data (clustering at the county level).

We computed 2 separate models. In the first model, we added patient race and ethnicity and patient’s DE status to evaluate their relative association with stroke severity. We tested for interaction between race and ethnicity and DE in the model. In the second model, we created 8 mutually exclusive categories: Black non-DE, Black DE, Hispanic non-DE, Hispanic DE, White non-DE, White DE, other non-DE, and other DE. We ran a multilevel multinomial logistic regression model using these 8 groups to examine the associations between these groups and stroke severity. This model was adjusted for patient attributes, comorbid conditions, stroke risk factors, behavioral risk factors for stroke, and county-level attributes. All statistical analyses were performed using SAS version 9.4 (SAS Institute). Statistical significance was set at *P* < .05, and all tests were 2-tailed.

## Results

**Figure 1.**
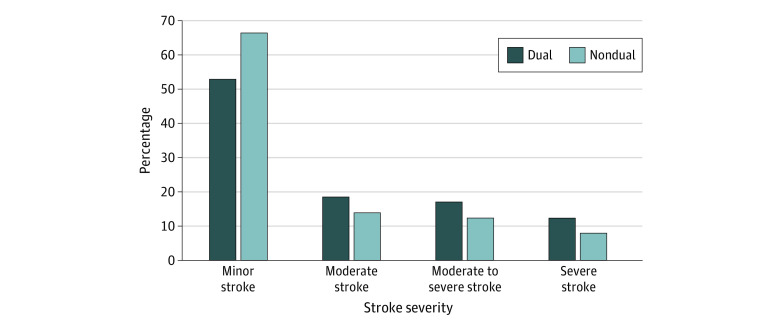
Association of Dual Eligibility With Stroke Severity Measured by National Institutes of Health Stroke Scale A score of 0 to 7 was considered minor; 8 to 13, moderate; 14-21, moderate to severe; and 22-42, severe.

**Figure 2.**
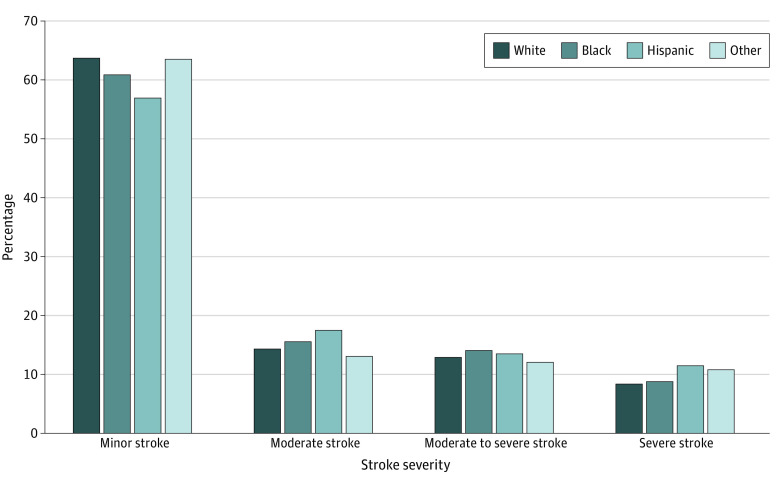
Association of Race and Ethnicity With Stroke Severity Measured by National Institutes of Health Stroke Scale A score of 0 to 7 was considered minor; 8 to 13, moderate; 14-21, moderate to severe; and 22-42, severe.

Our sample included 45 459 Medicare FFS patients, and 7738 (17.0%) of them were DE patients admitted with acute ischemic stroke. In our study cohort, the mean (SD) age was 80.2 (8.4) years; 25 303 (55.7%) were female; and 1719 (3.8%), Hispanic; 4107 (9.0%) were non-Hispanic Black; and 37 715 (83.0%) non-Hispanic Whites. Patient characteristics and social determinants of health by stroke severity are shown in Table 1. Patients with severe stroke were older (mean [SD] age, 83.3 [8.4] years) than patients with minor stroke (mean [SD] age 79.2 [8.2] years) and comprised a higher proportion of female compared with male patients (2606 [10.3%] vs 1371 [6.8%]). Patients with severe stroke had greater comorbidities indicated by the Elixhauser index score (mean [SD], 2.4 [2.1]) than patients with minor stroke (mean [SD], 1.0 [1.7]). Patients diagnosed with hypertension were more likely to have a severe stroke (8.9%) compared with patients without hypertension (6.4%). Finally, patients residing in areas with high poverty concentration were more likely to be in the higher stroke severity groups (9.4%) compared with patients in moderate (8.4%) or low poverty areas (8.6%). We have presented the distribution of NIHSS scores among our sample as a dependent variable by the primary independent variables of interest: DE status in Figure 1 and race and ethnicity in Figure 2. Among patients with DE, 52.6% had minor stroke compared with 66% of patients without DE, and patients with DE had a greater risk of more severe stroke than those without DE (Figure 1). Among White patients, 64.2% had minor strokes compared with 61.4% among Black patients and 57.4% among Hispanic patients (Figure 2). A higher proportion of minority patients were observed in the more severe stroke groups compared with White patients.

**Table 1.** Characteristics of the Study Population by Stroke Severity

Characteristic	Patients, No. (%)					*P* value
	All	Minor stroke, NIHSS score 0-7	Moderate stroke, NIHSS score 8-13	Moderate to severe stroke, NIHSS score 14-21	Severe stroke, NIHSS score 22-42	
**Demographic characteristics**						
Total	45 459 (100.0)	28 955 (63.7)	6570 (14.5)	5940 (13.1)	3994 (8.8)	NA
Age, mean (SD), y	80.2 (8.4)	79.2 (8.2)	81.0 (8.5)	82.0 (8.4)	83.3 (8.4)	<.001
Race and ethnicity						
Black	4107 (9.0)	2521 (61.4)	640 (15.6)	579 (14.1)	367 (8.9)	<.001
Hispanic	1719 (3.8)	986 (57.4)	301 (17.5)	232 (13.5)	200 (11.6)	
White	37 715 (83.0)	24 219 (64.2)	5378 (14.3)	4899 (13.0)	3219 (8.5)	
Other^a^	1918 (4.2)	1229 (64.1)	251 (13.1)	230 (12.0)	208 (10.8)	
Gender						
Male	20 156 (44.3)	13 818 (68.6)	2729 (13.5)	2230 (11.1)	1379 (6.8)	<.001
Female	25 303 (55.7)	15 137 (59.8)	3841 (15.2)	3710 (14.7)	2615 (10.3)	
Dual eligibility						
Dual	7738 (17.0)	4070 (52.6)	1407 (18.2)	1315 (17.0)	946 (12.2)	<.001
Nondual	37 721 (83.0)	24 885 (66.0)	5163 (13.7)	4625 (12.3)	3048 (8.1)	
Elixhauser Comorbidity Score, mean (SD)	45 459 (100.0)	1.0 (1.7)	1.6 (2.0)	2.0 (2.1)	2.4 (2.1)	<.001
Thrombectomy						
No	42 300 (93)	28 523 (67.4)	5966 (14.1)	4672 (11.0)	3139 (7.4)	<.001
Yes	3159 (7)	432 (13.7)	604 (19.1)	1268 (40.1)	855 (27.1)	
**Stroke Risk Factors**						
Hypertension						
No	1262 (2.8)	929 (73.6)	130 (10.3)	122 (9.7)	81 (6.4)	<.001
Yes	44 197 (97.2)	28 026 (63.4)	6440 (14.6)	5818 (13.2)	3913 (8.9)	
Hyperlipidemia						
No	3033 (6.7)	1788 (59.0)	399 (13.2)	499 (16.5)	347 (11.4)	<.001
Yes	42 426 (93.3)	27 167 (64.0)	6171 (14.6)	5441 (12.8)	3647(8.6)	
Diabetes						
No	23 294 (51.2)	14 979 (64.3)	3309 (14.2)	3050 (13.1)	1956 (8.4)	.005
Yes	22 165 (48.8)	13 976 (63.1)	3261 (14.7)	2890 (13.0)	2038 (9.2)	
Obesity						
No	32 298 (71.1)	20 423 (63.2)	4689 (14.5)	4259 (13.2)	2927 (9.1)	.002
Yes	13 161 (28.9)	8532 (64.8)	1881 (14.3)	1681 (12.8)	1067 (8.1)	
Alcohol use						.0004
No	42 511 (93.5)	27 086 (63.7)	6095 (14.3)	5542 (13.0)	3788 (8.9)	<.001
Yes	2948 (6.5)	1869 (63.4)	475 (16.1)	398 (13.5)	206 (7.0)	
Tobacco use						
No	37 373 (82.2)	23 633 (63.2)	5384 (14.4)	4947 (13.2)	3409 (9.1)	<.001
Yes	8086 (17.8)	5322 (65.8)	1186 (14.7)	993 (12.3)	585 (7.2)	
History of stroke						
No	31 095 (68.4)	20 246 (65.1)	4354 (14.0)	3956 (12.7)	2539 (8.2)	<.001
Yes	14 365 (31.6)	8709 (60.6)	2216 (15.4)	1984 (13.8)	1455 (10.1)	
**Social determinants of health by county**						
Poverty concentration						
Low	15 036 (33.1)	9738 (64.8)	2116 (14.1)	1892 (12.6)	1290 (8.6)	<.001
Moderate	15 356 (33.8)	9878 (64.3)	2162 (14.1)	2029 (13.2)	1287 (8.4)	
High	15 067 (33.1)	9339 (62.0)	2292 (15.2)	2019 (13.4)	1417 (9.4)	
Proportion of adults with high school or equivalent education						
Low	15 296 (33.7)	9914 (64.8)	2120 (13.9)	1997 (13.1)	1265 (8.3)	.001
Moderate	14 966 (32.9)	9529 (63.7)	2163 (14.5)	1958 (13.1)	1316 (8.8)	
High	15 197 (33.4)	9512 (62.6)	2287 (15.1)	1985 (13.1)	1413 (9.3)	
Concentration of physicians and nurse practitioners						
Low	15 153 (33.3)	9685 (63.9)	2190 (14.5)	1943 (12.8)	1335 (8.8)	.93
Moderate	15 099 (33.2)	9582 (63.5)	2202 (14.6)	2001 (13.3)	1314 (8.7)	
High	15 207 (33.5)	9688 (63.7)	2178 (14.3)	1996 (13.1)	1345 (8.8)	
Residing in stroke belt						
No	35 529 (78.2)	22 776 (64.1)	5082 (14.3)	4539 (12.8)	3132 (8.8)	<.001
Yes	9930 (21.8)	6179 (62.2)	1488 (15.0)	1401 (14.1)	862 (8.7)	

Abbreviations: NA, not applicable; NIHSS, National Institutes of Health Stroke Scale.

^a^ Individuals listed as Alaska Native, American Indian, Asian, Pacific Islander, other, or unknown were grouped under other for this analysis.

Most associations between race and ethnicity and stroke severity and all associations between DE and stroke severity from the multilevel multinomial regression model were significant after adjusting for patient clinical characteristics and social determinant of health factors at county level (Table 2). Using minor stroke in non-DE beneficiaries as the reference category, DE patients had 1.64 (95% CI, 1.45-1.86) times higher odds of having a moderate to severe stroke, and 1.73 (95% CI, 1.52-1.98) times higher odds of having a severe stroke than patients without DE. Additional analysis on estimates of all other covariates and interaction of race and ethnicity with DE status from the multilevel multinomial regression model are presented in eTable 1 in the Supplement.

**Table 2.** Association of Race and Ethnicity and DE Status With Stroke Severity in Medicare Patients With Ischemic Stroke^a^

Group	Patients, No. (%)	Moderate vs minor stroke, OR (95% CI)	*P* value	Moderate to severe vs minor stroke, OR (95% CI)	*P* value	Severe vs minor stroke, OR (95% CI)	*P* value
Non-DE	37 721 (83.0)	1 [Reference]	NA	1 [Reference]	NA	1 [Reference]	NA
DE	7738 (17.0)	1.38 (1.24-1.54)	<.001	1.64 (1.45-1.86)	<.001	1.73 (1.52-1.98)	<.001
White	37 715 (83.0)	1 [Reference]	NA	1 [Reference]	NA	1 [Reference]	NA
Black	4107 (9.0)	1.11 (1.00-1.23)	.06	1.15 (1.03-1.29)	.02	1.21 (1.06-1.39)	.006
Hispanic	1719 (3.8)	1.28 (1.11-1.47)	.001	1.12 (0.95-1.33)	.17	1.54 (1.29-1.85)	<.001
Other^b^	1918 (4.2)	0.93 (0.80-1.08)	.32	1.03 (0.87-1.21)	.77	1.50 (1.26-1.79)	<.001

Abbreviations: DE, dual eligibility; NA, not applicable; OR, odds ratio.

^a^ Model was adjusted for age, gender, race and ethnicity, DE status, interaction term between race and ethnicity and DE, Elixhauser Comorbidity Score, thrombectomy, stroke risk factors, prior history of stroke, social determinants of health at the county level, and an indicator for whether patient resides in the stroke belt. There was no significant interaction between race and ethnicity and DE (*P* value for interaction = .10). The estimates have been presented in eTable 1 in the Supplement.

^b^ Individuals listed as Alaska Native, American Indian, Asian, Pacific Islander, other, or unknown were grouped under other for this analysis.

After adjusting for age, sex, comorbidities, and stroke risk factors, and using minor stroke as the reference category, Black patients had 1.15 (95% CI, 1.03-1.29) times higher odds of having a moderate to severe stroke and 1.21 (95% CI, 1.06-1.39) times higher odds of having a severe stroke than White patients. Similarly, using minor stroke as the reference category, Hispanic patients had 1.28 (95% CI, 1.11-1.47) times higher odds of a moderate stroke and 1.54 (95% CI, 1.29-1.85) times higher odds of a severe stroke compared with White patients.

Additionally, to test for an interaction between race and ethnicity and DE status, we presented the results by 8 mutually exclusive groups (Table 3). Using White patients without DE as a reference, relative to the minor stroke category, White patients with DE had 1.79 (95% CI, 1.64-1.97) times higher odds of having a moderate to severe stroke and 1.75 (95% CI, 1.56-1.95) times higher odds of having a severe stroke. On the other hand, Black patients with DE had 1.94 (95% CI, 1.65-2.28) times higher odds of having a moderate to severe stroke and 2.15 (95% CI, 1.78-2.60) times higher odds of having a severe stroke than White patients without DE. Hispanic patients with DE had 1.95 (95% CI, 1.58-2.42) times higher odds of moderate to severe stroke and 2.50 (95% CI, 1.98-3.16) times higher odds of severe stroke than White patients without DE. Likewise, patients in other racial and ethnic groups with DE had 1.69 (95% CI, 1.34-2.14) times higher odds of moderate to severe stroke and 2.74 (95% CI, 2.16-3.47) times higher odds of severe stroke than White patients without DE.

**Table 3.** Association of Race and Ethnicity and Dual Eligibility Status With Stroke Severity in 8 Mutually Exclusive Groups in Medicare Patients With Ischemic Stroke^a^

Group	Patients, No.(%)	Moderate vs minor stroke, OR (95% CI)	*P* value	Moderate to severe vs minor stroke, OR (95% CI)	*P* value	Severe vs minor stroke, OR (95% CI)	*P* value
**White**							
Non-DE	32 984 (72.6)	1 [Reference]	NA	1 [Reference]	NA	1 [Reference]	NA
DE	4731 (10.4)	1.69 (1.55-1.84)	<.001	1.79 (1.64-1.97)	<.001	1.75 (1.56-1.95)	<.001
**Black**							
DE	2668 (5.9)	1.81 (1.56-2.10)	<.001	1.94 (1.65-2.28)	<.001	2.15 (1.78-2.60)	<.001
Non-DE	1439 (3.2)	1.14 (1.01-1.29)	.03	1.22 (1.07-1.40)	.003	1.19 (1.01-1.41)	.04
**Hispanic**							
DE	868 (1.9)	1.82 (1.50-2.21)	<.001	1.95 (1.58-2.42)	<.001	2.50 (1.98-3.16)	<.001
Non-DE	851 (1.9)	1.51 (1.25-1.83)	<.001	1.16 (0.92-1.47)	.22	1.67 (1.30-2.14)	<.001
**Other** ^b^							
DE	1201 (2.6)	1.28 (1.02-1.62)	.04	1.69 (1.34-2.14)	<.001	2.74 (2.16-3.47)	<.001
Non-DE	717 (1.6)	1.13 (0.95-1.35)	.18	1.11 (0.91-1.37)	.30	1.44 (1.14-1.82)	.002

Abbreviations: DE, dual eligibility; NA, not applicable; OR, odds ratio.

^a^ Model was adjusted for age, race and ethnicity, gender, DE status, Elixhauser Comorbidity Score, thrombectomy, stroke risk factors, prior history of stroke, social determinants of health at the county level, and an indicator for whether patient resides in the stroke belt.

^b^ Individuals listed as Alaska Native, American Indian, Asian, Pacific Islander, other, or unknown were grouped under other for this analysis.

### Sensitivity Analysis

To capture the association of delay in access to stroke care on stroke severity, we did sensitivity analyses on the distribution of NIHSS by process of admission types, transfers, and access to acute stroke care (eTable 2, eTable 3, and eTable 4 in the Supplement). There was no difference in stroke severity by adding the admission types, transfers, and access to acute stroke care in the model. Additionally, we also presented the distribution of NIHSS on postacute care and discharge destination (eTable 5 in the Supplement). We found that NIHSS severity is directly associated with intensity of postacute care, which is interesting, and further study is recommended to explore this area of research.

## Discussion

In this study using the NIHSS—a standardized, objective measure of stroke severity obtained from US Medicare population data—we found greater stroke severity among patients with DE, Black patients, and Hispanic patients compared with patients without DE and White patients, respectively, after accounting for stroke risk factors, comorbidities, and social risk factors. Most notably, the association of DE was more significant, and we found greater stroke severity among White patients with DE, Black patients with DE, and Hispanic patients with DE than among White patients without DE. As we know from past research, compared with patients without DE, those with DE are a diverse group of people who experience a higher number of chronic conditions and stroke risk factors (hypertension, hyperlipidemia, obesity, diabetes) than the general population. Individuals with DE are also less likely to receive preventive care and more likely to receive care in poor-quality hospitals and nursing homes.^5,38,39^ The greater stroke incidence found among patients with DE underscores the need for community-based stroke prevention programs for these individuals.^22^

In addition to stroke risk reduction programs, targeted efforts to improve poststroke outcomes through timely initiation of care should also be made by stroke centers and other hospitals serving DE and minority stroke patients. Recently, implementation of a prehospital triage program for patients with suspected large vessel occlusions directly to appropriate endovascular-capable stroke centers significantly improved rates of endovascular therapy, which is known to improve functional outcomes.^40^ Similarly, the use of mobile stroke units, even in smaller urban areas have shown to be effective in reducing door-to-needle time compared with traditional emergency transport.^41,42^ High-risk patients, patients with DE, and minority patients should be intentionally targeted by such programs. Furthermore, living in neighborhoods with high SES compared with low SES has been associated with better poststroke function, biopsychosocial health, and fewer depressive symptoms in adults with moderate to severe strokes as well as better function in those with minor strokes.^37^ Thus, in addition to receiving prompt intervention at primary and comprehensive stroke hospitals, DE should be discharged to inpatient rehabilitation facilities for comprehensive interdisciplinary postacute care. Our findings that patients with DE have more severe strokes than those without DE expands on previous work by suggesting that individuals with low SES experience disparities from the time of stroke occurrence.

The findings of this study affirm the presence of racial and ethnic disparities at stroke onset, which points to continued disparity in preventive stroke care, including prevention and management of hypertension, diabetes, and multiple vascular risk factors.^43^ Black individuals have a higher prevalence of stroke risk factors,^44^ which partially explains the higher incidence of stroke among Black adults compared with White adults and underscores the need for preventive care. Racial and ethnic disparities also affect hospital arrival times by patients after stroke,^45,46^ which may help to explain our findings of greater stroke severity among Black and Hispanic patients or patients with poor SES. Both mechanical thrombectomy and intravenous tissue plasminogen activator (tPA) have been shown to be used less with Black patients than with White patients.^47,48^ These interventions have a rapid positive effect on NIHSS scores, and improved 24-hour scores are predictive of long-term outcomes.^49,50^ In a largely Black, urban area, Black patients were one-third less likely to receive intravenous tPA in comparison with White patients, but they were also less likely to present within 3 hours of symptom onset. Of those who did present within 3 hours, almost half as many Black patients were likely to be treated with intravenous tPA than White patients.^47^ Each of these can contribute to worse NIHSS scores for patients.

Our results shows that DE status among White, Black, and Hispanic patients had a stronger association with stroke severity, affirming the implications of poor preventive care. This finding also helps to explain why DE and minority patients have worse long-term functional outcomes. Greater stroke severity at the time of diagnosis supports a need to focus more efforts on stroke prevention and early management of stroke in these vulnerable populations.

### Limitations and Strengths

This study has limitations. First, because we used Medicare claims data, we could not account for the timing of administration of the NIHSS during acute hospitalization. However, the American Heart Association and American Stroke Association strongly recommend hospitals document the first NIHSS within 12 hours of arrival at the acute hospital. Additionally, CMS recommends reporting the first NIHSS score within 12 hours of admission. However, at present, inpatient claims data do not have the timing of care; thus, we do not know whether patients received intervention before NIHSS documentation, and the reported NIHSS could be the result of the intervention provided. Second, hospitals began reporting NIHSS scores after October 1, 2016, but rural hospitals still do not report NIHSS scores.^51^ Therefore, our findings cannot be generalized to rural hospitals. Third, administrative claims data may have inaccurate coding and potential missing data, although high levels of agreement have been reported between administrative data and electronic medical records.^52,53,54^ Fourth, inpatient claims information for Medicare Advantage (MA) patients are still missing in current Medicare data. Since missing NIHSS scores in MA patients could induce bias in our study, we excluded MA patients. Therefore, our results may not apply to patients enrolled in MA plans.

Aside from these limitations, there are some strengths to our work. Understanding variation in stroke care is crucial, and patient-level clinical risk factors do not account for all observed disparities in stroke severity. Our study addressed limitations of previous studies by adjusting for both patient-level stroke risk factors and social determinant of health variables at the county level, minimizing the risk of confounding present in the absence of risk factors.

## Conclusions

This study found that patients with DE, Black patients, and Hispanic patients with ischemic stroke had more severe strokes than their non-DE and White counterparts. Of note, DE status had a compounding association with stroke severity regardless of race and ethnicty, including among White patients, which has immense implications on Medicare policies for improving preventive care and stroke care. Although disparity in stroke is one of the most compelling public health issues in the United States, our findings indicate that DE status, a proxy for low SES, and race and ethnicity are associated with stroke severity. Not only to minimize stroke severity, but to address the current health care crisis in the United States, researchers should identify community-level interventions designed to reduce disparities in access to preventive, early, and postacute stroke care for people with low SES and racial and ethnic minority individuals. It is crucial that patients with DE, Black patients, and Hispanic patients receive timely care in stroke-certified hospitals to ensure receipt of interventions that have been most effective in achieving equitable outcomes.

## Notes

Supplement. **eTable 1.** Estimates of All Other Covariates From Multilevel Multinomial Regression Model **eTable 2.** Distribution of NIHSS by Process of Admission Types, Transfers, and Access to Acute-Stroke Care **eTable 3.** Risk Adjusted Odds of Stroke Severity by Race and Ethnicity and Dual Eligibility Status After Including Admission Types, Transfers, and Procedure of Access to Acute-Stroke Care **eTable 4.** Risk Adjusted Odds of Stroke Severity in 8 Mutually Exclusive Groups by Race and Ethnicity and Dual Eligibility After Including Admission Types, Transfers, and Procedure of Access to Acute-Stroke Care **eTable 5.** Distribution of Postacute Care and Discharge Destination by Stroke Severity **eFigure.** Cohort Derivation
