# Error in Abstract and Methods

**DOI:** 10.1001/jamanetworkopen.2022.12579

**Published:** 2022-04-21

In the Original Investigation titled “Association of Medicare-Medicaid Dual Eligibility and Race and Ethnicity With Ischemic Stroke Severity,”^1^ published March 31, 2022, there were errors in the Abstract and Methods section. In both places, the range of dates for Medicare data capture should have been reported as October 1, 2016, to December 31, 2017. This article has been corrected.^1^
