# Supplementary material for: Association of Medicare-Medicaid Dual Eligibility and Race and Ethnicity With Ischemic Stroke Severity
Source: JAMA Netw Open. 2022 Mar 31;5(3):e224596. doi: 10.1001/jamanetworkopen.2022.4596 (PMC8972034; doi:10.1001/jamanetworkopen.2022.4596)
Supplement: Supplement. — eTable 1. Estimates of All Other Covariates From Multilevel Multinomial Regression Model eTable 2. Distribution of NIHSS by Process of Admission Types, Transfers, and Access to Acute-Stroke Care eTable 3. Risk Adjusted Odds of Stroke Severity by Race and Ethnicity and Dual Eligibility Status After Including Admission Types, Transfers, and Procedure of Access to Acute-Stroke Care eTable 4. Risk Adjusted Odds of Stroke Severity in 8 Mutually Exclusive Groups by Race and Ethnicity and Dual Eligibility After Including Admission Types, Transfers, and Procedure of Access to Acute-Stroke Care eTable 5. Distribution of Postacute Care and Discharge Destination by Stroke Severity eFigure. Cohort Derivation [file jamanetwopen-e224596-s001.pdf]

Supplemental Online Content

Bosch PR, Karmarkar AM, Roy I, Fehnel CR, Burke RE, Kumar A. Association of Medicare-Medicaid dual eligibility and race and ethnicity with ischemic stroke severity. *JAMA Netw Open.* 2022;5(3):e224596. doi:10.1001/jamanetworkopen.2022.4596

eTable 1. Estimates of All Other Covariates From Multilevel Multinomial Regression Model

eTable 2. Distribution of NIHSS by Process of Admission Types, Transfers, and Access to Acute-Stroke Care

eTable 3. Risk Adjusted Odds of Stroke Severity by Race and Dual Eligibility Status After Including Admission Types, Transfers, and Procedure of Access to Acute-Stroke Care

eTable 4. Risk Adjusted Odds of Stroke Severity in 8 Mutually Exclusive Groups by Race and Ethnicity and Dual Eligibility After Including Admission Types, Transfers, and Procedure of Access to Acute-Stroke Care

eTable 5. Distribution of Postacute Care and Discharge Destination by Stroke Severity

eFigure. Cohort Derivation

This supplemental material has been provided by the authors to give readers additional information about their work.

© 2022 Bosch PR et al. *JAMA Network Open.*

eTable 1: Estimates of All Other Covariates From Multilevel Multinomial Regression Model

	Stroke Severity (Odds Ratios from multinomial regression)		
	**Moderate vs Minor Stroke OR (95% CI) (p-value)**	**Moderate to Severe vs Minor Stroke OR (95% CI) (p-value)**	**Severe vs Minor Stroke OR (95% CI) (p-value)**
**Race/Ethnicity (Reference: White)**			
Black	1.11 (1.00 - 1.23) (0.06)	1.15 (1.03 - 1.29) (0.02)	1.21 (1.06 - 1.39) (0.006)
Hispanic	1.28 (1.11 - 1.47) (0.001)	1.12 (0.95 - 1.33) (0.17)	1.54 (1.29 - 1.85) (<.0001)
Others	0.93 (0.80 - 1.08) (0.32)	1.03 (0.87 - 1.21) (0.77)	1.50 (1.26 - 1.79) (<.0001)
**Dual Eligibility (Reference: Non-DE)**			
DE	1.38 (1.24 - 1.54) (<.0001)	1.64 (1.45 - 1.86) (<.0001)	1.73 (1.52 -1.98) (<.0001)
**Estimates from Interaction of Race/Ethnicity and Dual Eligibility**			
(Black vs White for DE) vs (Black vs White for Non-DE)	0.94 (0.77 - 1.14) (0.51)	0.88 (0.71 - 1.10) (0.27)	1.04 (0.80 - 1.34) (0.80)
(Hispanic vs White for DE) vs (Hispanic vs White for non-DE)	0.72 (0.54 - 0.95) (0.02)	0.94 (0.68 - 1.30) (0.70)	0.86 (0.61 - 1.22) (0.40)
(Other vs White for DE) vs (Others vs White for non-DE)	0.67 (0.50 - 0.91) (0.01)	0.85 (0.62 - 1.16) (0.30)	1.09 (0.78 - 1.53) (0.63)
**Gender (Reference: Male)**			
Female	1.14 (1.08 - 1.21) (<.0001)	1.26 (1.18 - 1.34) (<.0001)	1.33 (1.23 - 1.43) (<.0001)
**Stroke Risk Factors (Reference: No for all)**			
Hypertension	1.30 (1.07 - 1.57) (0.01)	1.22 (0.99 - 1.50) (0.07)	1.05 (0.82 - 1.35) (0.69)
Hyperlipidemia	0.90 (0.80- 1.02) (0.09)	0.60 (0.54 – 0.68) (<.0001)	0.54 (0.48 – 0.68) (<.0001)
Diabetes	1.01 (0.95 - 1.07) (0.72)	1.01 (0.95 - 1.08) (0.70)	1.09 (1.01 - 1.18) (0.02)
Obesity	0.99	1.03	0.98

© 2022 Bosch PR et al. *JAMA Network Open.*

	(0.93 - 1.06)<br>(0.81)	(0.96 - 1.11)<br>(0.37)	(0.91 - 1.07)<br>(0.71)
Alcohol Use	1.20<br>(1.08 - 1.34)<br>(0.001)	1.18<br>(1.04 - 1.34)<br>(0.009)	0.97<br>(0.82 - 1.14)<br>(0.69)
Tobacco use	1.05<br>(0.97 - 1.13)<br>(0.24)	1.04<br>(0.96 - 1.14)<br>(0.31)	0.99<br>(0.89 - 1.10)<br>(0.83)
**Thrombectomy (Reference: No)**			
Yes	7.16<br>(6.30 - 8.14)<br>(<.0001)	20.10<br>(17.86 - 22.63)<br>(<.0001)	21.45<br>(18.86 - 24.40)<br>(<.0001)
**History of Stroke (Reference: No)**			
Yes	1.08<br>(1.01 - 1.14)<br>(0.02)	1.07<br>(1.00 - 1.14)<br>(0.04)	1.17<br>(1.08 - 1.26)<br>(<.0001)
**Social Determinants of Health by County**			
Poverty Concentration (Reference: Low)			
Medium	0.98<br>(0.91 - 1.05)<br>(0.14)	1.05<br>(0.96 - 1.14)<br>(0.30)	0.95<br>(0.85 - 1.05)<br>(0.32)
High	1.06<br>(0.98 - 1.14)<br>(0.96)	1.05<br>(0.96 - 1.14)<br>(0.34)	1.06<br>(0.95 - 1.18)<br>(0.31)
Concentration of Physicians and Nurse Practitioners (Reference: High)			
Low	1.02<br>(0.95 - 1.10)<br>(0.56)	0.98<br>(0.90 - 1.07)<br>(0.64)	0.98<br>(0.88 - 1.09)<br>(0.67)
Medium	1.02<br>(0.95 - 1.10)<br>(0.59)	1.00<br>(0.92 - 1.09)<br>(0.93)	0.97<br>(0.88 - 1.08)<br>(0.58)
Proportion with High School or Equivalent Education (Reference: Low)			
Medium	1.06<br>(0.99 - 1.14)<br>(0.11)	1.04<br>(0.95 - 1.14)<br>(0.42)	1.15<br>(1.03 - 1.29)<br>(0.02)
High	1.17<br>(1.09 - 1.26)<br>(<.0001)	1.11<br>(1.01 - 1.21)<br>(0.03)	1.36<br>(1.22 - 1.52)<br>(<.0001)
**Residing in Stroke Belt (Reference: No)**			
Yes	1.06<br>(0.99 - 1.14)<br>(0.09)	1.14<br>(1.05 - 1.24)<br>(0.002)	1.03<br>(0.93 - 1.14)<br>(0.58)
Model was adjusted for- Age, race, gender, dual eligibility (DE), Elixhauser, thrombectomy, stroke risk factors, prior history of stroke, social determinants of health at the county level and an indicator for whether patient resides in the stroke belt.			

© 2022 Bosch PR et al. *JAMA Network Open.*

eTable 2. Distribution of NIHSS by Process of Admission Types, Transfers, and Access to Acute-Stroke Care

**Variables**	**N (% of Total)**	**NIHSS 0-7 (Minor Stroke)**	**NIHSS 8-13 (Moderate Stroke)**	**NIHSS 14-21 (Moderate to Severe Stroke)**	**NIHSS 22-42 (Severe Stroke)**	**P value**
Physician/Clinic Referral	37,147 (81.7)	66.3	14.0	12.0	7.7	<.0001
Transferred from Hospital	7,108 (15.6)	53.3	15.8	17.5	13.4	
Transferred from SNF	1,132 (2.5)	43.0	20.1	20.9	16.0	
Ambulatory	7 (0.02)	28.6	14.3	42.9	14.3	
Unknown	65 (0.14)	56.9	15.4	18.5	9.2	

Note: The variable TRNSFR_CD indicates the source of the referral for the admission.

© 2022 Bosch PR et al. *JAMA Network Open.*

eTable 3. Risk Adjusted Odds of Stroke Severity by Race and Dual Eligibility Status After Including Admission Types, Transfers, and Procedure of Access to Acute-Stroke Care

	Stroke Severity (Odds Ratios from multinomial regression)		
	**Moderate vs Minor Stroke OR 95% CI**	**Moderate to Severe vs Minor Stroke OR 95% CI**	**Severe vs Minor Stroke OR 95% CI**
**Difference by Dual Eligibility Status (Reference: Non-Duals)**			
DE	1.37 (1.23 - 1.53)	1.62 (1.44 - 1.84)	1.71 (1.49 -1.96)
**Difference by Race/Ethnicity (Reference: White)**			
Black	1.13 (1.02 - 1.25)	1.18 (1.06 - 1.33)	1.26 (1.10 - 1.45)
Hispanic	1.30 (1.12 - 1.49)	1.15 (0.97 - 1.35)	1.59 (1.33 - 1.90)
Others	0.95 (0.81 - 1.10)	1.05 (0.90 - 1.24)	1.56 (1.31 - 1.85)
This model controls all covariates as the original model, along with the additional covariate admission types and transfers. De= dual eligibility			

© 2022 Bosch PR et al. *JAMA Network Open.*

eTable 4. Risk Adjusted Odds of Stroke Severity in 8 Mutually Exclusive Groups by Race and Ethnicity and Dual Eligibility After Including Admission Types, Transfers, and Procedure of Access to Acute-Stroke Care

	Moderate vs Minor Stroke OR (95% CI)	Moderate to Severe vs Minor Stroke OR (95% CI)	Severe vs Minor Stroke OR (95% CI)
**Reference: White-Non-DE**			
White DE	1.65 (1.51 - 1.80)	1.74 (1.58 - 1.91)	1.69 (1.51 - 1.89)
Black DE	1.81 (1.56 - 2.10)	1.95 (1.66 - 2.29)	2.18 (1.81 - 2.63)
Black Non-DE	1.16 (1.03 - 1.31)	1.25 (1.10 - 1.43)	1.24 (1.05 - 1.46)
Hispanic DE	1.82 (1.50 - 2.20)	1.95 (1.57 - 2.41)	2.50 (1.97 - 3.16)
Hispanic Non-DE	1.52 (1.26 - 1.84)	1.18 (0.93 - 1.49)	1.71 (1.33 - 2.19)
Other DE	1.30 (1.03 - 1.64)	1.73 (1.37 - 2.19)	2.83 (2.23 - 3.59)
Other Non-DE	1.13 (0.95 - 1.36)	1.11 (0.91 - 1.37)	1.44 (1.14 - 1.83)
This model controls all covariates as the original model, along with the additional covariate admission types and transfers. DE= dual eligibility			

© 2022 Bosch PR et al. *JAMA Network Open.*

eTable 5. Distribution of Postacute Care and Discharge Destination by Stroke Severity

Variables	N (%)	NIHSS 0-7 (Minor Stroke)	NIHSS 8-13 (Moderate Stroke)	NIHSS 14-21 (Moderate to Severe Stroke)	NIHSS 22-42 (Severe Stroke)	P value
Home	11,518 (26.8)	89.1	6.7	3.2	1.0	<.0001
Home with Home Health	5,562 (12.9)	81.4	10.2	6.0	2.4	
Skilled Nursing Facilities	9,990 (23.2)	55.2	19.4	16.6	8.8	
Inpatient Rehabilitation Facilities	11,471 (26.7)	62.0	19.6	13.4	5.0	
Hospice	3,052 (7.1)	16.9	15.6	32.7	34.8	
Others	1,415 (3.3)	50.0	17.5	19.3	13.2	

© 2022 Bosch PR et al. *JAMA Network Open.*

eFigure. Cohort Derivation

Cohort Derivation

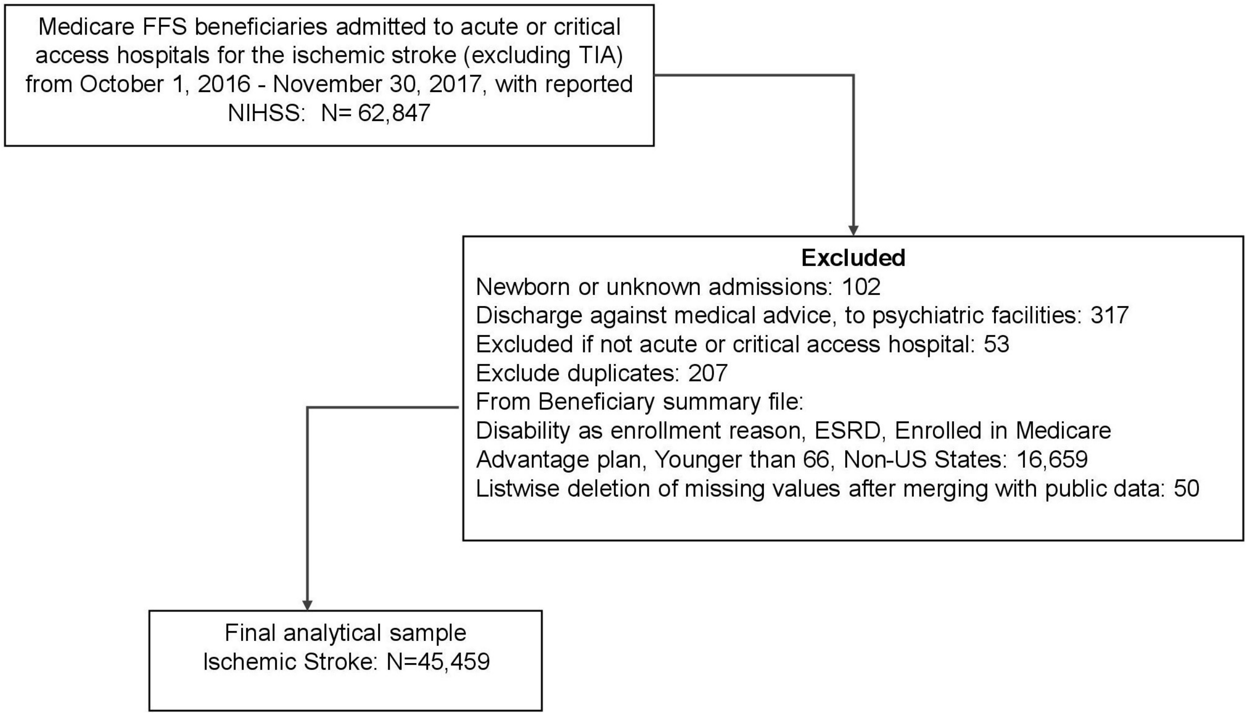

© 2022 Bosch PR et al. *JAMA Network Open.*
